# Robust joint analysis allowing for model uncertainty in two-stage genetic association studies

**DOI:** 10.1186/1471-2105-12-9

**Published:** 2011-01-07

**Authors:** Dongdong Pan, Qizhai Li, Ningning Jiang, Aiyi Liu, Kai Yu

**Affiliations:** 1 Department of Statistics, Yunnan University, Kunming 650091, PR China; 2 Academy of Mathematics and Systems Science, Chinese Academy of Sciences, Beijing 100190, PR China; 3 Biostatistics and Bioinformatics Branch, Eunice Kennedy Shriver National Institute of Child Health and Human Development, Bethesda, MD 20892, USA; 4 Biostatistics Branch, Division of Cancer Epidemiology and Genetics, National Cancer Institute, Bethesda, MD 20892, USA

## Abstract

**Background:**

The cost efficient two-stage design is often used in genome-wide association studies (GWASs) in searching for genetic loci underlying the susceptibility for complex diseases. Replication-based analysis, which considers data from each stage separately, often suffers from loss of efficiency. Joint test that combines data from both stages has been proposed and widely used to improve efficiency. However, existing joint analyses are based on test statistics derived under an assumed genetic model, and thus might not have robust performance when the assumed genetic model is not appropriate.

**Results:**

In this paper, we propose joint analyses based on two robust tests, MERT and MAX3, for GWASs under a two-stage design. We developed computationally efficient procedures and formulas for significant level evaluation and power calculation. The performances of the proposed approaches are investigated through the extensive simulation studies and a real example. Numerical results show that the joint analysis based on the MAX3 test statistic has the best overall performance.

**Conclusions:**

MAX3 joint analysis is the most robust procedure among the considered joint analyses, and we recommend using it in a two-stage genome-wide association study.

## Background

The two-stage design is often adopted in genome-wide association studies (GWASs) to search for genetic variants underlying susceptibility for complex diseases. The advantages of the two-stage design have been investigated extensively (see *e.g*., [1-12]). In a typical two-stage design for GWASs, a proportion of the available samples are genotyped at the initial stage on a large number of single nucleotide polymorphisms (SNPs) using a commercial genotyping platform. Based on association test results obtained at this stage, a small percentage of SNPs are selected and further genotyped on the remaining samples in the second stage. To analyze data generated from such a two-stage design, the joint analysis strategy has been recommended, which combines the test statistics from both stages as the final test statistic, and is shown to be more powerful than the replication-based analysis that only utilizes the second stage data [12].

The efficiency of joint analysis based on the allele-frequency-difference-based test (AFDT) was evaluated in detail in comparison to the replication-based analysis [12]. It is commonly adopted as a single marker test in GWASs. The AFDT is valid when Hardy-Weinberg equilibrium (HWE) holds in the target population, and is powerful when the underlying genetic models are additive or multiplicative. The Cochran-Armitage trend test (CATT) [13,14] derived under the additive (in log scale) genetic risk model is also used in single-maker analysis, which is optimal when the underlying additive genetic model is true. However, both tests are not so powerful compared with other methods such as MAX3 [15] when the underlying genetic model is not additive. Since in most cases there is no evidence suggesting that the additive risk model is most appropriate for the underlying disease model, especially in the typical GWASs where we most likely evaluate only the tagging SNPs, but not the causal SNPs directly. Thus, it is advantageous to adopt a more robust single marker test that has a relatively good performance under all possible disease models. To this end, two types of such robust tests, the MERT (maximin efficiency robust test) [15,16] and MAX3 (the maximum values of CATTs under recessive, additive and dominant models) have been recently considered [15,17]. Nevertheless, their performances under the two-stage design have not been thoroughly investigated.

In this report we propose two types of joint test statistics for the two-stage design based on the two robust tests, MERT and MAX3. We derive closed-form formula to calculate the power of the MERT-based joint analysis, and propose a computationally efficient Monte Carlo procedure to evaluate the significance level of the MAX3-based joint analysis. Facilitated by these two procedures, we evaluate the performances of the two robust test based joint analyses, in comparison with the ones based on AFDT, under various two-stage design setups and disease models.

## Methods

### Notations

Suppose that *r *cases and *s *controls are randomly sampled from the source population in a GWAS. Denote the number of SNPs genotyped and the proportion of the subjects in Stage 1 by *m *and *π*, respectively. Throughout, we only consider biallelic SNPs with two alleles G and g, with G being the risk allele. Then there are three genotypes: gg, Gg, and GG. Using the disease risk at gg as the baseline, we define the relative risks of Gg and GG as *λ*_1 _= *f*_1_/*f*_0 _and *λ*_2 _= *f*_2_/*f*_0_, respectively, where *f*_0 _= Pr(*case*|*gg*) > 0, *f*_1 _= Pr(*case*|*Gg*), *f*_2 _= Pr(*case*|*GG*) are the penetrances. Let *K *= Pr(*case*) be the disease prevalence. Denote the genotype frequencies in case population as *p*_0 _= Pr(*gg*|*case*) = Pr(*gg*)*f*_0_/*K*, *p*_1 _= Pr(*Gg*|*case*) = Pr(*Gg*)*f*_1_/*K*, *p*_2 _= Pr(*GG*|*case*) = Pr(*GG*)*f*_2_/*K *and in control population as *q*_0 _= Pr(*gg*|*control*) = Pr(*gg*)(1-*f*_0_)/(1-*K*), *q*_1 _= Pr(*Gg*|*control*) = Pr(*Gg*)(1-*f*_1_)/(1-*K*), *q*_2 _= Pr(*GG*|*control*) = Pr(*GG*)(1-*f*_2_)/(1-*K*). Then the null hypothesis of no association is *H*_0 _: *p_i _*= *q_i_*, *i *= 0, 1, 2, which is equivalent to *H*_0 _: *λ*_1 _= *λ*_2 _= 1. The alternative hypothesis is *H*_1 _: *λ*_2 _≥ *λ*_1 _≥ 1 with *λ*_2 _> 1. The commonly used three genetic models, recessive, additive and dominant models are corresponding to *λ*_2 _>*λ*_1 _= 1, 2*λ*_1 _= *λ*_2 _+1 and *λ*_1 _= *λ*_2 _> 1, respectively. We assume that SNPs with *p*-values less than *γ *in Stage 1 will be further investigated in Stage 2 and α be the whole genome-wide type I error.

The notations for genotype frequencies in case population and control population of Stage 1 and Stage 2 are given in Table 1. It should be noted that *p*_1*i *_= *p*_2*i *_and *q*_1*i *_= *q*_2*i *_for *i *= 0, 1, 2 in the table using the first subscript on behalf of Stage 1 or Stage 2 since they are the population parameters. However, the estimates of *p*_1*i *_and *q*_1*i *_for *i *= 0, 1, 2 based on the data of Stage 1 and those of *p*_2*i *_and *q*_2*i *_for *i *= 0, 1, 2 based on the data of Stage 2 might be different although the data of Stage 1 and Stage 2 are drawn from the same source population.

**Table 1:** Genotype frequencies in case population and control population for both stages

	cases			controls		
	gg	Gg	GG	gg	Gg	GG
Stage 1	*p*_10_	*p*_11_	*p*_12_	*q*_10_	*q*_11_	*q*_12_
Stage 2	*p*_20_	*p*_21_	*p*_22_	*q*_20_	*q*_21_	*q*_22_

### Allele-Frequency-Difference-Based Joint Analysis

Denote the risk allele frequencies in case population and control population by *θ *and ϖ, respectively. Let ${\hat{\theta }}_{1}$ and ${\hat{\varpi }}_{1}$ be their maximum likelihood estimates in Stage 1, respectively. Then the test statistic for Stage 1 is

$$\begin{array}{l} Z_{1}=\frac{{\hat{\theta }}_{1}-{\hat{\varpi }}_{1}}{\sqrt{\frac{1}{2r\pi }+\frac{1}{2s\pi }}}\\ \times \frac{1}{\sqrt{\left[{\hat{\theta }}_{1}\xi +{\hat{\varpi }}_{1}(1-\xi )\right]\left[1-{\hat{\theta }}_{1}\xi -{\hat{\varpi }}_{1}(1-\xi )\right]}},\text{ where }\xi =\frac{r}{r+s}. \end{array}$$

The threshold for selecting SNPs in Stage 1 is *b*_1 _= Φ^-1^(1-*γ*/2), where Φ(·) is the cumulative standard normal distribution function. Similarly, we can get the maximum likelihood estimates of the risk allele frequencies in case population and control population using the data from Stage 2, denoted by ${\hat{\theta }}_{2}$ and ${\hat{\varpi }}_{2}$. Then the test statistic for Stage 2 can be written as

$$\begin{array}{l} Z_{2}=\frac{{\hat{\theta }}_{2}-{\hat{\varpi }}_{2}}{\sqrt{\frac{1}{2r(1-\pi )}+\frac{1}{2s(1-\pi )}}}\\ \times \frac{1}{\sqrt{\left[{\hat{\theta }}_{2}\xi +{\hat{\varpi }}_{2}(1-\xi )\right]\left[1-{\hat{\theta }}_{2}\xi -{\hat{\varpi }}_{2}(1-\xi )\right]}}. \end{array}$$

The joint statistic is $Z_{J}=\sqrt{\pi }Z_{1}+\sqrt{1-\pi }Z_{2}$. The Bonferroni correction threshold (*b_J_*) for *Z_J _*is the solution of the equation ${\mathrm{Pr}}_{{\text{H}}_{\text{0}}}\left(\left|{\text{Z}}_{\text{1}}\right|>b_{1},\left|Z_{J}\right|>b_{J}\right)=\alpha /m$,

where ${(Z_{1},Z_{J})'|}_{{\text{H}}_{\text{0}}}\sim N_{2}((0,0)',\Gamma \Gamma ')$, $\Gamma =\left(\begin{array}{cc} 1 & 0\\ \sqrt{\pi } & \sqrt{1-\pi } \end{array}\right)$. So the power of the joint test under the alternative hypothesis is given by ${\mathrm{Pr}}_{{\text{H}}_{\text{1}}}\left(\left|{\text{Z}}_{\text{1}}\right|>b_{1},\left|Z_{J}\right|>b_{J}\right)$, where ${\left(\begin{array}{c} Z_{1}\\ Z_{J} \end{array}\right)|}_{{\text{H}}_{\text{1}}}\sim N_{2}\left(\left(\begin{array}{c} \mu_{1}\\ \sqrt{\pi }\mu_{1}+\sqrt{1-\pi }\mu_{2} \end{array}\right),\Gamma \Delta_{1}\Gamma '\right)$, with $\begin{array}{l} \mu_{1}=\frac{\theta_{1}-\varpi_{1}}{\sqrt{\frac{1}{2r\pi }+\frac{1}{2s\pi }}}\\ \times \frac{1}{\sqrt{\left[\theta_{1}\xi +\varpi_{1}(1-\xi )\right]\left[1-\theta_{1}\xi -\varpi_{1}(1-\xi )\right]}} \end{array}$, $\begin{array}{l} \mu_{2}=\frac{\theta_{2}-\varpi_{2}}{\sqrt{\frac{1}{2r(1-\pi )}+\frac{1}{2s(1-\pi )}}}\\ \times \frac{1}{\sqrt{\left[\theta_{2}\xi +\varpi_{2}(1-\xi )\right]\left[1-\theta_{2}\xi -\varpi_{2}(1-\xi )\right]}} \end{array}$, $\Delta_{1}=\left(\begin{array}{cc} \delta_{1} & 0\\ 0 & \delta_{2} \end{array}\right)$, $\delta_{1}=\frac{\left(1-\xi )\theta_{1}(1-\theta_{1})+\xi \varpi_{1}(1-\varpi_{1}\right)}{\left[\theta_{1}\xi +\varpi_{1}(1-\xi )\right]\left[1-\theta_{1}\xi -\varpi_{1}(1-\xi )\right]}$ and $\delta_{2}=\frac{\left(1-\xi )\theta_{2}(1-\theta_{2})+\xi \varpi_{2}(1-\varpi_{2}\right)}{\left[\theta_{2}\xi +\varpi_{2}(1-\xi )\right]\left[1-\theta_{2}\xi -\varpi_{2}(1-\xi )\right]}$.

The calculation of ${\mathrm{Pr}}_{{\text{H}}_{\text{1}}}\left(\left|{\text{Z}}_{\text{1}}\right|>b_{1},\left|Z_{J}\right|>b_{J}\right)$ is based on two-fold integration which can be computed using the built-in function, "pmvnorm", in the R package "mvtnorm" [18-20].

The above approach is slightly different from the one considered in [12], where the authors constructed the test statistics by estimating the variance of the differences of allele frequency between case population and control population using the cases and controls separately under the null hypothesis. In our joint analysis, we estimated the variance using the combined data of case sample and control sample. Results (not show here) show that the two approaches have very similar performance.

### Cochran-Armitage Trend Test under the Additive Model-Based Joint Analysis

Cochran-Armitage trend test under the additive model (CATTA) (see *e.g.*, [13,15]) is often used in the genetic association studies including GWASs. Denote CATTA for both stages by $T_{1}^{A}$ and $T_{2}^{A}$, respectively. Then the threshold for selecting SNPs in Stage 1 is *d*_1 _= Φ^-1^(1-*γ*/2). The joint test statistic is $T_{J}^{A}=\sqrt{\pi }T_{1}^{A}+\sqrt{1-\pi }T_{2}^{A}$. The threshold (*d_J_*) for $T_{J}^{A}$ can be obtained by solving the equation ${\mathrm{Pr}}_{{\text{H}}_{\text{0}}}\left(\left|T_{1}^{A}\right|>d_{1},\left|T_{J}^{A}\right|>d_{J}\right)=\alpha /m$. The power of the joint analysis is ${\mathrm{Pr}}_{{\text{H}}_{\text{1}}}\left(\left|T_{1}^{A}\right|>d_{1},\left|T_{J}^{A}\right|>d_{J}\right)$, which can be calculated again using the R package "mvtnorm". The joint distributions of $\left(T_{1}^{A},T_{J}^{A}\right)'$ under the null and alternative hypotheses are given in Appendix A in Additional file 1.

### MERT-Based Joint Analysis

MERT was originally proposed in [16] to find robust test statistic in situations when multiple alternative models are plausible. It was used to define a robust test for single-marker analysis [15]. Here we apply the test to two-stage design. Similar to $T_{1}^{A}$ and $T_{2}^{A}$, we can obtain CATTs $T_{1}^{R}$ and $T_{2}^{R}$ under the recessive model and CATTs $T_{1}^{D}$ and $T_{2}^{D}$ under the dominant model for both stages. So MERT for both stages are $T_{1}^{mert}=\frac{T_{1}^{R}+T_{1}^{D}}{{\left[2(1+\rho_{1}^{RD})\right]}^{1/2}}$ and $T_{2}^{mert}=\frac{T_{2}^{R}+T_{2}^{D}}{{\left[2(1+\rho_{2}^{RD})\right]}^{1/2}}$, respectively, where $\rho_{1}^{RD}$ and $\rho_{2}^{RD}$ are the correlation coefficients of $T_{1}^{R}$ and $T_{1}^{D}$, and $T_{2}^{A}$ and $T_{2}^{D}$ under the null hypothesis, respectively, which are shown in Appendix B in Additional file 1. The joint analysis based on MERT can be defined as $T_{J}^{mert}=\sqrt{\pi }T_{1}^{mert}+\sqrt{1-\pi }T_{2}^{mert}$. The threshold for selecting SNPs in Stage 1 is *u*_1 _= Φ^-1^(1-*γ*/2). To control the false positive rate of the joint analysis, we can obtain the threshold *u_J_*, which is the solution to the equation

$${\mathrm{Pr}}_{{\text{H}}_{\text{0}}}\left(\left|T_{1}^{mert}\right|>u_{1},\left|T_{J}^{mert}\right|>u_{J}\right)=\alpha /m.$$

The power of the test is given by ${\mathrm{Pr}}_{{\text{H}}_{\text{1}}}\left(\left|T_{1}^{mert}\right|>u_{1},\left|T_{J}^{mert}\right|>u_{J}\right)$, whose numerical values can be calculated using the R package "mvtnorm". The joint distributions of $\left(T_{1}^{mert},T_{J}^{mert}\right)'$ under the null and alternative hypotheses are derived in Appendix B in Additional file 1.

### MAX3-Based Joint Analysis

MAX3, the maximal value of CATT under three genetic models, is another commonly used robust test in the current GWASs (see *e.g.*, [7,15,17]). Once we have $\left(T_{1}^{R},T_{1}^{A},T_{1}^{D}\right)$ and $\left(T_{2}^{R},T_{2}^{A},T_{2}^{D}\right)$, the test statistic in Stage 1 is $T_{1}^{\max}=\max\left\{\left|T_{1}^{R}\right|,\left|T_{1}^{A}\right|,\left|T_{1}^{D}\right|\right\}$ and the joint analysis based on MAX3 can be defined as $T_{J}^{\max}=\max\left\{\left|T_{J}^{R}\right|,\left|T_{J}^{A}\right|,\left|T_{J}^{D}\right|\right\}$, where $T_{J}^{R}=\sqrt{\pi }T_{1}^{R}+\sqrt{1-\pi }T_{2}^{R}$, $T_{J}^{A}=\sqrt{\pi }T_{1}^{A}+\sqrt{1-\pi }T_{2}^{A}$, and $T_{J}^{D}=\sqrt{\pi }T_{1}^{D}+\sqrt{1-\pi }T_{2}^{D}$. For a given significance level γ in Stage 1, the threshold (*v*_1_) can be obtained by solving the equation ${\mathrm{Pr}}_{{\text{H}}_{\text{0}}}\left(\max\left\{\left|T_{1}^{R}\right|,\left|T_{1}^{A}\right|,\left|T_{1}^{D}\right|\right\}>v_{1}\right)=\gamma$.

According to Chapter 6 of [21], we have $T_{1}^{A}=\omega_{11}T_{1}^{R}+\omega_{12}T_{1}^{D}$, where $\omega_{11}=\frac{\rho_{1}^{RA}-\rho_{1}^{RD}\rho_{1}^{AD}}{1-{\left(\rho_{1}^{RD}\right)}^{2}}$ and $\omega_{12}=\frac{\rho_{1}^{AD}-\rho_{1}^{RD}\rho_{1}^{RA}}{1-{\left(\rho_{1}^{RD}\right)}^{2}}$, with $\rho_{1}^{RA}$ and $\rho_{1}^{AD}$ given in Appendix C in Additional file 1. Because $\left(\begin{array}{c} T_{1}^{R}\\ T_{1}^{D} \end{array}\right)|{}_{H_{0}}\sim N_{2}\left(\left(\begin{array}{c} 0\\ 0 \end{array}\right),\left(\begin{array}{cc} 1 & \rho_{1}^{RD}\\ \rho_{1}^{RD} & 1 \end{array}\right)\right)$, we can obtain *v*_1 _using the R package "mvtnorm". After that, we use the following computationally efficient algorithm to approximate the threshold (*v_J_*) for the joint analysis:

1) Generate *B *identical and independently distributed bivariate normal random variates $\left(T_{11}^{R},T_{11}^{D}\right)',\left(T_{12}^{R},T_{12}^{D}\right)',\cdots ,\left(T_{1B}^{R},T_{1B}^{D}\right)'\sim N_{2}\left(\left(\begin{array}{c} 0\\ 0 \end{array}\right),\left(\begin{array}{cc} 1 & \rho_{1}^{RD}\\ \rho_{1}^{RD} & 1 \end{array}\right)\right)$. Then calculate $T_{1i}^{A}=\omega_{\text{11}}T_{1i}^{R}+\omega_{12}T_{1i}^{D}$, and $T_{1i}^{\max}=\max\left\{\left|T_{1i}^{R}\right|,\left|T_{1i}^{A}\right|,\left|T_{1i}^{D}\right|\right\}$ for *i *= 1, 2, ⋯, *B*. Without loss of generality, we assume $T_{1i}^{\max}>v_{1}$ for *i *= 1, 2, ⋯, *B*_1 _and $T_{1i}^{\max}\leq v_{1}$ for *i *= *B*_1 _+ 1, *B*_1 _+ 2, ⋯. *B*.

2) Generate *B*_1 _identical and independently distributed bivariate normal random variates $\left(T_{21}^{R},T_{21}^{D}\right)',\left(T_{22}^{R},T_{22}^{D}\right)',\cdots ,\left(T_{2B_{1}}^{R},T_{2B_{1}}^{D}\right)'\sim N_{2}\left(\left(\begin{array}{c} 0\\ 0 \end{array}\right),\left(\begin{array}{cc} 1 & \rho_{2}^{RD}\\ \rho_{2}^{RD} & 1 \end{array}\right)\right)$. Then calculate $T_{2i}^{A}=\omega_{\text{21}}T_{2i}^{R}+\omega_{22}T_{2i}^{D}$, $\omega_{21}=\frac{\rho_{2}^{RA}-\rho_{2}^{RD}\rho_{2}^{AD}}{1-{\left(\rho_{2}^{RD}\right)}^{2}}$ and $\omega_{22}=\frac{\rho_{2}^{AD}-\rho_{2}^{RD}\rho_{2}^{RA}}{1-{\left(\rho_{2}^{RD}\right)}^{2}}$, with $\rho_{2}^{RA}$ and $\rho_{2}^{AD}$ given in Appendix C in Additional file 1. For *i *= 1, 2, ⋯, *B*_1_, calculate

$$T_{Ji}^{\max}=\max\left\{\begin{array}{l} \left|\sqrt{\pi }T_{1i}^{R}+\sqrt{1-\pi }T_{2i}^{R}\right|,\\ \left|\sqrt{\pi }T_{1i}^{A}+\sqrt{1-\pi }T_{2i}^{A}\right|,\\ \left|\sqrt{\pi }T_{1i}^{D}+\sqrt{1-\pi }T_{2i}^{D}\right| \end{array}\right\}.$$

3) Find *v_J _*that yields $\min\left|\frac{\#\left\{T_{Ji}^{\max}>v_{J},i=1,2,\cdots ,B_{1}\right\}}{B_{1}}-\frac{\alpha }{m\gamma }\right|$ with $\frac{\#\left\{T_{Ji}^{\max}>v_{J},i=1,2,\cdots ,B_{1}\right\}}{B_{1}}\leq \frac{\alpha }{m\gamma }$ and $v_{J}\in \left\{T_{Ji}^{\max},i=1,2,\cdots ,B_{1}\right\}$.

Once we have *v*_1 _and *v_J_*, we generate the data under the alternative hypothesis to calculate the power empirically. In the simulation studies, we generate 10,000 data sets under the alternative hypothesis. For the *i*^th ^data set (*i *= 1, 2. ⋯, 10000), we calculate $T_{1}^{\max}$ and $T_{J}^{\max}$, denote them again by $T_{1i}^{\max}$ and $T_{Ji}^{\max}$, respectively. Then the empirical power is $\frac{\#\left\{T_{1i}^{\max}>v_{1},T_{Ji}^{\max}>v_{J};i=1,2,\cdots ,10000\right\}}{10000}$.

## Results

### Simulation Setup

In order to mimic the real GWAS, we choose the simulation parameters similar to [12,22]. In a typical GWAS, there are thousands of individuals randomly chosen from the source population and the number of SNPs being examined in Stage 1 is usually from 0.1 million to 1 million. Based on the results of Stage 1 (*p*-values), the number of SNPs to be genotyped in Stage 2 is in tens or hundreds. For example, in a diabetes mellitus GWAS [7], there were 392,935 SNPs genotyped on 1,363 subjects in Stage 1, and 57 SNPs were genotyped in Stage 2 after removing those SNPs with *p*-values greater than 0.0001 based on the data of Stage 1. In a GWAS, the significance level in the whole genome is often set to be 0.05, and the Bonferroni-correction is often used to adjust for multiple comparisons and to control the false positive rate. So, in our simulation studies, we set the number of SNPs at Stage 1 *m *= 500,000 and the *p*-value threshold for significant SNPs to be 0.05/*m *= 1 ×10^-7^. The proportion of subjects genotyped in Stage 1 is set to be 0.5, 0.4 and 0.3, and the *p*-value threshold for SNPs selection at the end of Stage 1 be 0.0001 and 0.0002. The disease prevalence is set to be *K *= 0.1. Throughout our simulation procedures, we assume that Hardy-Weinberg equilibrium (HWE) holds in the general population. Furthermore, the risk allele is assumed to be the minor allele, with frequency (MAF) equal to 0.15, 0.25, 0.35 and 0.45. The considered genetic models are the recessive, additive, and dominant models. We specified different genotype relative risks *λ*_1 _and *λ*_2 _for the three genetic models (see details in Table 2, 3, 4 and 5). The critical values for MAX3 joint analysis are simulated, while thresholds for other three joint analysis are exactly calculated based on their asymptotic distributions under the null hypothesis where the genotype probabilities (*p*_0_, *p*_1_, *p*_2_) for cases and *(q*_0_, *q*_1_, *q*_2_) for controls are calculated by *p*_0 _= *q*_0 _= Pr(*gg*) = (1-MAF)^2^, *p*_1 _= *q*_1 _= Pr(*Gg*) = 2 × MAF ×(1-MAF) and *p*_2 _= *q*_2 _= Pr(*GG*) = MAF^2^. Under the alternative hypothesis, the genotype frequencies can be obtained using the formulas given in the Notations Subsection and *f*_0 _= *K*/[Pr(*gg*) + *λ*_1 _Pr(*Gg*) + *λ*_2 _Pr(*GG*)]. More details could be referred to [23] and [24]. The genotype counts in case sample and control sample were generated from a multinomial distribution.

**Table 2:** Power comparison for MAF = 0.15 (K = 0.1, α = 0.05, m = 5 × 10^5^)

	π	γ	ALLEJ	CATAJ	MERTJ	MAX3J
Recessive Model	0.5	0.0001	0.070	0.058	0.385	0.759
*r *= *s *= 5000		0.0002	0.076	0.064	0.420	0.798
*λ*_1 _= 1, *λ*_2 _= 2	0.4	0.0001	0.049	0.042	0.273	0.583
		0.0002	0.058	0.048	0.317	0.646
	0.3	0.0001	0.029	0.025	0.155	0.346
		0.0002	0.036	0.031	0.193	0.423

Additive Model	0.5	0.0001	0.601	0.613	0.440	0.555
*r *= *s *= 2000		0.0002	0.643	0.655	0.477	0.599
*λ*_1 _= 1.4, *λ*_2 _= 1.8	0.4	0.0001	0.450	0.460	0.317	0.406
		0.0002	0.507	0.517	0.364	0.451
	0.3	0.0001	0.271	0.277	0.183	0.226
		0.0002	0.326	0.334	0.226	0.281

Dominant Model	0.5	0.0001	0.679	0.711	0.356	0.726
*r *= *s *= 2000		0.0002	0.720	0.752	0.388	0.768
*λ*_1 _= *λ*_2 _= 1.5	0.4	0.0001	0.520	0.551	0.254	0.552
		0.0002	0.579	0.611	0.293	0.621
	0.3	0.0001	0.322	0.345	0.146	0.339
		0.0002	0.383	0.408	0.181	0.400

**Table 3:** Power comparison for MAF = 0.25 (K = 0.1, α = 0.05, m = 5 × 10^5^)

	π	γ	ALLEJ	CATAJ	MERTJ	MAX3J
Recessive Model	0.5	0.0001	0.075	0.066	0.220	0.517
*r *= *s *= 5000		0.0002	0.083	0.073	0.242	0.546
*λ*_1 _= 1, *λ*_2 _= 1.5	0.4	0.0001	0.053	0.047	0.154	0.365
		0.0002	0.062	0.055	0.180	0.408
	0.3	0.0001	0.031	0.028	0.087	0.197
		0.0002	0.039	0.035	0.110	0.254

Additive Model	0.5	0.0001	0.835	0.846	0.782	0.799
*r *= *s *= 2000		0.0002	0.868	0.878	0.820	0.838
*λ*_1 _= 1.4, *λ*_2 _= 1.8	0.4	0.0001	0.687	0.700	0.625	0.639
		0.0002	0.742	0.754	0.683	0.700
	0.3	0.0001	0.462	0.474	0.405	0.413
		0.0002	0.530	0.542	0.472	0.476

Dominant Model	0.5	0.0001	0.717	0.757	0.511	0.826
*r *= *s *= 2000		0.0002	0.758	0.796	0.551	0.853
*λ*_1 _= *λ*_2 _= 1.5	0.4	0.0001	0.557	0.597	0.375	0.651
		0.0002	0.617	0.656	0.427	0.726
	0.3	0.0001	0.350	0.382	0.221	0.425
		0.0002	0.413	0.447	0.270	0.495

**Table 4:** Power comparison for MAF = 0.35 (K = 0.1, α = 0.05, m = 5 × 10^5^)

	π	γ	ALLEJ	CATAJ	MERTJ	MAX3J
Recessive Model	0.5	0.0001	0.420	0.384	0.536	0.824
*r *= *s *= 4000		0.0002	0.456	0.418	0.578	0.860
*λ*_1 _= 1, *λ*_2 _= 1.5	0.4	0.0001	0.302	0.274	0.393	0.657
		0.0002	0.348	0.317	0.447	0.717
	0.3	0.0001	0.175	0.158	0.231	0.436
		0.0002	0.216	0.196	0.282	0.492

Additive Model	0.5	0.0001	0.891	0.900	0.882	0.864
*r *= *s *= 2000		0.0002	0.916	0.925	0.909	0.895
*λ*_1 _= 1.4, *λ*_2 _= 1.8	0.4	0.0001	0.760	0.773	0.747	0.715
		0.0002	0.809	0.821	0.797	0.767
	0.3	0.0001	0.537	0.551	0.522	0.481
		0.0002	0.604	0.618	0.590	0.548

Dominant Model	0.5	0.0001	0.558	0.607	0.464	0.758
*r *= *s *= 2000		0.0002	0.600	0.649	0.502	0.806
*λ*_1 _= *λ*_2 _= 1.5	0.4	0.0001	0.413	0.455	0.337	0.599
		0.0002	0.468	0.512	0.385	0.660
	0.3	0.0001	0.246	0.274	0.197	0.374
		0.0002	0.298	0.330	0.242	0.437

**Table 5:** Power comparison for MAF = 0.45 (K = 0.1, α = 0.05, m = 5 × 10^5^)

	π	γ	ALLEJ	CATAJ	MERTJ	MAX3J
Recessive Model	0.5	0.0001	0.282	0.253	0.263	0.542
*r *= *s *= 2000		0.0002	0.308	0.277	0.288	0.572
*λ*_1 _= 1, *λ*_2 _= 1.5	0.4	0.0001	0.199	0.178	0.184	0.380
		0.0002	0.231	0.207	0.215	0.442
	0.3	0.0001	0.114	0.101	0.104	0.220
		0.0002	0.142	0.127	0.131	0.263

Additive Model	0.5	0.0001	0.886	0.896	0.894	0.854
*r *= *s *= 2000		0.0002	0.912	0.921	0.919	0.881
*λ*_1 _= 1.4, *λ*_2 _= 1.8	0.4	0.0001	0.753	0.767	0.765	0.701
		0.0002	0.803	0.815	0.813	0.760
	0.3	0.0001	0.529	0.543	0.542	0.473
		0.0002	0.597	0.610	0.609	0.545

Dominant Model	0.5	0.0001	0.279	0.317	0.302	0.590
*r *= *s *= 2000		0.0002	0.305	0.346	0.329	0.626
*λ*_1 _= *λ*_2 _= 1.5	0.4	0.0001	0.197	0.225	0.214	0.428
		0.0002	0.229	0.260	0.248	0.483
	0.3	0.0001	0.112	0.128	0.123	0.241
		0.0002	0.140	0.160	0.153	0.291

### Simulation Results

For convenience, we refer to the aforementioned four joint analysis approaches as, respectively, ALLEJ (allele-frequency-difference-based joint analysis), CATAJ (Cochran-Armitage trend test under the additive model-based joint analysis), MERTJ (MERT-based joint analysis), MAX3J (MAX3-based joint analysis). Table 2, 3, 4 and 5 report powers of the four joint analysis methods corresponding to MAF equal to 0.15, 0.25, 0.35 and 0.45, respectively. From these tables, we have the following observations. Under the recessive model, MERTJ and MAX3J are more powerful than ALLEJ and CATAJ, with MAX3J being most powerful among the four methods under consideration. In some cases, the advantage of MAX3J is quite impressive. For example, in Table 2, with *π *= 0.5, *γ *= 0.0001, the powers of ALLEJ, CATAJ, MERTJ and MAX3J are 0.070, 0.058, 0.385 and 0.759, respectively. Under the additive model, CATAJ and ALLEJ have comparable power and are more powerful than the other two tests. However, the power difference between CATAJ and MAX3J is mostly at the level of 6.6%, with the largest discrepancy of 7%. Under the dominant model, CATAJ and MAX3J are more powerful than ALLEJ and MERTJ. Both tests have comparable power when MAF = 0.15, and MAX3J is much more powerful than CATAJ when MAF = 0.25, 0.35 and 0.45. In summary, it appears that MAX3J has the best overall performance.

### A Real Example: Type 2 Diabetes Mellitus

Type 2 diabetes mellitus is one of the most common diseases, and has been found to be associated with environmental factors and genetic variants. A two-stage GWAS for type 2 diabetes mellitus was reported in [7]. In this study, 392,935 SNPs were genotyped on 1,363 subjects in Stage 1. Based on the statistical significance level of 1 × 10^-4^, 57 SNPs were selected and further screened on 2,617 cases and 2,894 controls in Stage 2. We applied the above four considered methods to two SNPs, rs1005316 and rs2876711, which were not reported in their Table 1, but were shown in their Appendix. Table 6 gives the genotype counts and *p*-values of these two SNPs. We found a genome-wide significant association between rs2876711 and the outcome. Although the association between rs2876711 and type 2 diabetes mellitus has not been reported by [7], our results show that we should be concerned with this SNP and its neighborhood area. Additional experiments should be further conducted to validate this association.

**Table 6:** Genotype counts and *p*-values of SNPs rs1005316 and rs2876711 for type 2 diabetes mellitus

SNP ID		r_0_	r_1_	r_2_	s_0_	s_1_	s_2_	ALLEJ	CATAJ	MERTJ	MAX3J
rs1005316	Stage 1	13	224	457	44	211	399	6.13 × 10^-5^	7.78 × 10^-6^	3.87 × 10^-6^	8.12 × 10^-7^
	Stage 2	89	669	1708	89	913	1856				
rs2876711	Stage 1	99	322	272	121	351	182	2.92 × 10^-7^	2.07 × 10^-8^	5.97 × 10^-8^	3.10 × 10^-8^
	Stage 2	389	1191	989	484	1404	987				

*Note: *r_0_, r_1_, and r_2 _denote the number of individuals carrying genotype gg, Gg, and GG in case sample, respectively; s_0_, s_1_, and s_2 _denote the number of individuals carrying genotype gg, Gg, and GG in control sample, respectively.

## Discussion and Conclusions

In genetic association studies, the underlying genetic inheritance model is often unknown, and thus hinders the use of methods such as CATT, which has to be derived under an assumed genetic model. Robust tests, such as MERT and MAX3, had been proposed to relax the dependence on the underlying genetic models. Extending these tests to a two-stage setting, we construct two robust joint analyses based on MERT and MAX3. Numerical results show that MAX3J has the best overall performance among the four considered joint analysis approaches. For type 2 diabetes mellitus, based on MAX3J, we found that SNP rs2876711 was significantly associated with type 2 diabetes mellitus besides their findings.

Pearson Chi-square test is a robust test that was used in genetic association studies (see *e.g.*, [25]). Recently, a comprehensive power comparison between MAX3 and Pearson Chi-square test and Cochran-Armitage trend test under the additive model was conducted in [17]. They reported that MAX3 has the most robust performances. The proposed joint analysis combing the test statistics of both stages considers the between-stage heterogeneity. It is intractable for Pearson Chi-square test to consider the relative risk heterogeneity of both stages, especially when the relative risk in Stage 1 is larger than one and that in Stage 2 is less than one.

Recently, a joint analysis based on genetic model selection [26] to overcome the genetic model uncertainty was proposed in [22]. Based on the data in Stage 1, they used Hardy-Weinberg disequilibrium trend test studied in [27] to determine a score that corresponds to a genetic model. This score was then used to construct the trend test based on the data of Stage 2. Results (not shown here) show that the proposed joint analysis has comparable power. Therefore, the proposed MAX3J can be used as an alternative procedure in two-stage genome-wide association studies.

## Abbreviations

GWAS: genome-wide association study; SNP: single nucleotide polymorphism; MAF: minor allele frequency; AFDT: allele-frequency-difference-based test; CATT: Cochran-Armitage trend test; MERT: maximin efficiency robust test; MAX3: maximum values of Cochran-Armitage trend tests under recessive, additive and dominant models; ALLEJ: allele-frequency-difference-based joint analysis; CATAJ: Cochran-Armitage trend test under the additive model-based joint analysis; MERTJ: MERT-based joint analysis; MAX3J: MAX3-based joint analysis.

## Authors' contributions

PD and LQ implemented the model. All authors read and approved the final manuscript.

## Supplementary Material

Additional file 1 **Appendix for the main text**. The file (including Appendix A, B, C) is a Microsoft Word document. Appendix A gives a detailed description of the joint distribution of the additive trend test statistic $T_{1}^{A}$ in Stage 1 and the joint additive trend test statistic $T_{J}^{A}$. Appendix B gives a detailed description of the correlation coefficient between the recessive trend test statistic and the dominant trend test statistic under the null hypothesis, and the joint distribution of $T_{1}^{mert}$ and $T_{J}^{mert}$. Appendix C gives a detailed description of the correlation coefficient between the recessive trend test statistic and the additive trend test statistic, and the correlation coefficient between the additive trend test statistic and the dominant trend test statistic. Click here for file
