# Supplementary material for: Robust joint analysis allowing for model uncertainty in two-stage genetic association studies
Source: BMC Bioinformatics. 2011 Jan 7;12:9. doi: 10.1186/1471-2105-12-9 (PMC3027114; doi:10.1186/1471-2105-12-9)
Supplement: Additional file 1 — Appendix for the main text. The file (including Appendix A, B, C) is a Microsoft Word document. Appendix A gives a detailed description of the joint distribution of the additive trend test statistic T1A in Stage 1 and the joint additive trend test statistic TJA. Appendix B gives a detailed description of the correlation coefficient between the recessive trend test statistic and the dominant trend test statistic under the null hypothesis, and the joint distribution of T1mert and TJmert. Appendix C gives a detailed description of the correlation coefficient between the recessive trend test statistic and the additive trend test statistic, and the correlation coefficient between the additive trend test statistic and the dominant trend test statistic. [file 1471-2105-12-9-S1.DOC]

**Robust joint analysis allowing for model uncertainty in two-stage**

**genetic association studies**

Dongdong Pan, Qizhai Li, Ningning Jiang, Aiyi Liu, Kai Yu

**Appendix**

**Appendix A: The joint distribution of $\left(T_{1}^{A},T_{J}^{A}\right)'$.**

Note that in a two-stage GWAS, the population parameters $p_{1i}$, $q_{1i}$, $p_{2i}$ and $q_{2i}$,$i=0,1,2$, which are given in Table 1 in the main text, can be estimated based on the genotype frequencies in case sample and control sample of Stage 1 and Stage 2. We have

$\left.\left(T_{1}^{A},T_{J}^{A}\right)'\right|_{\text{H}_{0}}\sim N_{2}\left((0,0)',\Gamma\Gamma'\right)$.

$\left.\left(T_{1}^{A},T_{J}^{A}\right)'\right|_{\text{H}_{1}}\sim N_{2}\left(\left(\mu_{1}^{A},\sqrt{\pi}\mu_{1}^{A}+\sqrt{1-\pi}\mu_{2}^{A}\right)',\Gamma\Delta_{2}\Gamma'\right)$, where

$\mu_{1}^{A}=\frac{0.5(p_{11}-q_{11})+(p_{12}-q_{12})}{\left\{\left[0.25p_{11}+p_{12}-(0.5p_{11}+p_{12})^{2}\right]/(r\pi)+\left[0.25q_{11}+q_{12}-(0.5q_{11}+q_{12})^{2}\right]/(s\pi)\right\}^{1/2}}$,

$\mu_{2}^{A}=\frac{0.5(p_{21}-q_{21})+(p_{22}-q_{22})}{\left\{\left[0.25p_{21}+p_{22}-(0.5p_{21}+p_{22})^{2}\right]/\left[r(1-\pi)\right]+\left[0.25q_{21}+q_{22}-(0.5q_{21}+q_{22})^{2}\right]/\left[s(1-\pi)\right]\right\}^{1/2}},$and $\Delta_{2}=\left(\begin{matrix} \eta_{1}^{A} & 0 \\ 0 & \eta_{2}^{A} \end{matrix}\right)$, where

$\eta_{1}^{A}=\frac{(1-\xi)[0.25p_{11}+p_{12}-(0.5p_{11}+p_{12})^{2}]+\xi[0.25q_{11}+q_{12}-(0.5q_{11}+q_{12})^{2}]}{0.25(\xi p_{11}+(1-\xi)q_{11})+\xi p_{12}+(1-\xi)q_{12}-[0.5(\xi p_{11}+(1-\xi)q_{11})+\xi p_{12}+(1-\xi)q_{12}]^{2}}$,

$\eta_{2}^{A}=\frac{(1-\xi)[0.25p_{21}+p_{22}-(0.5p_{21}+p_{22})^{2}]+\xi[0.25q_{21}+q_{22}-(0.5q_{21}+q_{22})^{2}]}{0.25(\xi p_{21}+(1-\xi)q_{21})+\xi p_{22}+(1-\xi)q_{22}-[0.5(\xi p_{21}+(1-\xi)q_{21})+\xi p_{22}+(1-\xi)q_{22}]^{2}}$.

**Appendix B: $\rho_{1}^{RD}$ and $\rho_{2}^{RD}$ under the null hypothesis and the joint distribution of $\left(T_{1}^{mert},T_{J}^{mert}\right)'$.**

Using the notations in the main text, from Zheng and Gastwirth (2006) (see [14] in the References Section in the main text), the general formulas of the correlation coefficient between $T_{1}^{R}$ and $T_{1}^{D}$, and $T_{2}^{R}$ and $T_{2}^{D}$ are, respectively,

$\text{corr}(T_{1}^{R},T_{1}^{D})=\frac{(1-\xi)p_{10}p_{12}+\xi q_{10}q_{12}}{\left[(1-\xi)p_{10}(1-p_{10})+\xi q_{10}(1-q_{10})\right]^{1/2}\left[(1-\xi)p_{12}(1-p_{12})+\xi q_{12}(1-q_{12})\right]^{1/2}}$, and

$\text{corr}(T_{2}^{R},T_{2}^{D})=\frac{(1-\xi)p_{20}p_{22}+\xi q_{20}q_{22}}{\left[(1-\xi)p_{20}(1-p_{20})+\xi q_{20}(1-q_{20})\right]^{1/2}\left[(1-\xi)p_{22}(1-p_{22})+\xi q_{22}(1-q_{22})\right]^{1/2}}$.

Then $\rho_{1}^{RD}\text{=corr}(T_{1}^{R},T_{1}^{D})$, where $p_{1i}=q_{1i},i=0,1,2$, and $\rho_{2}^{RD}\text{=corr}(T_{2}^{R},T_{2}^{D})$, where $p_{2i}=q_{2i},i=0,1,2$.

Under the null hypothesis, the joint distribution of $\left(T_{1}^{mert},T_{J}^{mert}\right)'$ follows a bivariate normal distribution with mean $(0,0)'$ and variance-covariance matrix $\Gamma\Gamma'$, that is,

$\left.\left(T_{1}^{mert},T_{J}^{mert}\right)'\right|_{\text{H}_{0}}=\left.\Gamma\left(T_{1}^{mert},T_{2}^{mert}\right)'\right|_{\text{H}_{0}}\sim N_{2}\left((0,0)',\Gamma\Gamma'\right)$.

Define

$\mu_{1}^{R}=\frac{p_{12}-q_{12}}{[p_{12}(1-p_{12})/(r\pi)+q_{12}(1-q_{12})/(s\pi)]^{1/2}}$,

$\mu_{2}^{R}=\frac{p_{22}-q_{22}}{\left\{[p_{22}(1-p_{22})/[r(1-\pi)]+q_{22}(1-q_{22})/[s(1-\pi)]\right\}^{1/2}}$,

$\mu_{1}^{D}=\frac{(p_{11}-q_{11})+(p_{12}-q_{12})}{\left\{[p_{11}+p_{12}-(p_{11}+p_{12})^{2}]/(r\pi)+[q_{11}+q_{12}-(q_{11}+q_{12})^{2}]/(s\pi)\right\}^{1/2}}$, and

$\mu_{2}^{D}=\frac{(p_{21}-q_{21})+(p_{22}-q_{22})}{\left\{[p_{21}+p_{22}-(p_{21}+p_{22})^{2}]/[r(1-\pi)]+[q_{21}+q_{22}-(q_{21}+q_{22})^{2}]/[s(1-\pi)]\right\}^{1/2}}$.

$\eta_{1}^{R}=\frac{(1-\xi)[p_{12}(1-p_{12})]+\xi[q_{12}(1-q_{12})]}{\xi p_{12}+(1-\xi)q_{12}-[\xi p_{12}+(1-\xi)q_{12}]^{2}}$,

$\eta_{2}^{R}=\frac{(1-\xi)[p_{22}(1-p_{22})]+\xi[q_{22}(1-q_{22})]}{\xi p_{22}+(1-\xi)q_{22}-[\xi p_{22}+(1-\xi)q_{22}]^{2}}$,

$\eta_{1}^{D}=\frac{(1-\xi)[p_{11}+p_{12}-(p_{11}+p_{12})^{2}]+\xi[q_{11}+q_{12}-(q_{11}+q_{12})^{2}]}{\xi p_{11}+(1-\xi)q_{11}+\xi p_{12}+(1-\xi)q_{12}-[\xi p_{11}+(1-\xi)q_{11}+\xi p_{12}+(1-\xi)q_{12}]^{2}}$, and

$\eta_{2}^{D}=\frac{(1-\xi)[p_{21}+p_{22}-(p_{21}+p_{22})^{2}]+\xi[q_{21}+q_{22}-(q_{21}+q_{22})^{2}]}{\xi p_{21}+(1-\xi)q_{21}+\xi p_{22}+(1-\xi)q_{22}-[\xi p_{21}+(1-\xi)q_{21}+\xi p_{22}+(1-\xi)q_{22}]^{2}}$.

$\sigma_{1}=\frac{\eta_{1}^{R}+\eta_{1}^{D}+2\text{corr}\left(T_{1}^{R},T_{1}^{D}\right)\sqrt{\eta_{1}^{R}\eta_{1}^{D}}}{2(1+\rho_{1}^{RD})}$,

$\sigma_{2}=\frac{\eta_{2}^{R}+\eta_{2}^{D}+2\text{corr}\left(T_{2}^{R},T_{2}^{D}\right)\sqrt{\eta_{2}^{R}\eta_{2}^{D}}}{2(1+\rho_{2}^{RD})}$,

$\mu_{1}^{mert}=\frac{\mu_{1}^{R}+\mu_{1}^{D}}{\sqrt{2(1+\rho_{1}^{RD})}}$,

$\mu_{2}^{mert}=\frac{\mu_{2}^{R}+\mu_{2}^{D}}{\sqrt{2(1+\rho_{2}^{RD})}}$, and

$\Delta_{3}=\left(\begin{matrix} \sigma_{1} & 0 \\ 0 & \sigma_{2} \end{matrix}\right)$.

Then

$\left.\left(T_{1}^{mert},T_{J}^{mert}\right)'\right|_{\text{H}_{1}}=\left.\Gamma\left(T_{1}^{mert},T_{J}^{mert}\right)'\right|_{\text{H}_{1}}\sim N_{2}\left(\left(\mu_{1}^{mert},\sqrt{\pi}\mu_{1}^{mert}+\sqrt{1-\pi}\mu_{2}^{mert}\right),\Gamma\Delta_{3}\Gamma'\right)$.

**Appendix C: $\rho_{1}^{RA}$, $\rho_{1}^{AD}$, $\rho_{2}^{RA}$ and $\rho_{2}^{AD}$.**

Using the notations in the main text, from the results in Zheng and Gastwirth (2006) (see [14] in the References Section in the main text), we have

$\text{corr}(T_{1}^{R},T_{1}^{A})=\frac{(1-\xi)p_{12}(2p_{10}+p_{11})+\xi q_{12}(2q_{10}+q_{11})}{\sqrt{\delta_{10}}\sqrt{\delta_{11}}}$,

$\text{corr}(T_{1}^{A},T_{1}^{D})=\frac{(1-\xi)p_{10}(2p_{12}+p_{11})+\xi q_{10}(2q_{12}+q_{11})}{\sqrt{\delta_{11}}\sqrt{\delta_{12}}}$,

$\text{corr}(T_{2}^{R},T_{2}^{A})=\frac{(1-\xi)p_{22}(2p_{20}+p_{21})+\xi q_{22}(2q_{20}+q_{21})}{\sqrt{\delta_{20}}\sqrt{\delta_{21}}}$, and

$\text{corr}(T_{2}^{A},T_{2}^{D})=\frac{(1-\xi)p_{20}(2p_{22}+p_{21})+\xi q_{20}(2q_{22}+q_{21})}{\sqrt{\delta_{21}}\sqrt{\delta_{22}}}$,

where

$\delta_{10}=(1-\xi)p_{12}(1-p_{12})+\xi q_{12}(1-q_{12})$,

$\delta_{11}=(1-\xi)(p_{10}p_{11}+p_{11}p_{12}+4p_{10}p_{12})+\xi(q_{10}q_{11}+q_{11}q_{12}+4q_{10}q_{12})$,

$\delta_{12}=(1-\xi)p_{10}(1-p_{10})+\xi q_{10}(1-q_{10})$,

$\delta_{20}=(1-\xi)p_{22}(1-p_{22})+\xi q_{22}(1-q_{22})$,

$\delta_{21}=(1-\xi)(p_{20}p_{21}+p_{21}p_{22}+4p_{20}p_{22})+\xi(q_{20}q_{21}+q_{21}q_{22}+4q_{20}q_{22})$, and

$\delta_{22}=(1-\xi)p_{20}(1-p_{20})+\xi q_{20}(1-q_{20})$.

Therefore,

$\rho_{1}^{RA}\text{=corr}(T_{1}^{R},T_{1}^{A})$, where $p_{1i}=q_{1i},i=0,1,2$;

$\rho_{1}^{AD}\text{=corr}(T_{1}^{A},T_{1}^{D})$, where $p_{1i}=q_{1i},i=0,1,2$;

$\rho_{2}^{RA}\text{=corr}(T_{2}^{R},T_{2}^{A})$, where $p_{2i}=q_{2i},i=0,1,2$; and

$\rho_{2}^{AD}\text{=corr}(T_{2}^{A},T_{2}^{D})$, where $p_{2i}=q_{2i},i=0,1,2$.
